# Surgical Anatomy of the Glenoid Cavity and Its Use in Shoulder Arthroplasty Among the North Indian Population

**DOI:** 10.7759/cureus.11940

**Published:** 2020-12-06

**Authors:** Rajani Singh

**Affiliations:** 1 Anatomy, Uttar Pradesh University of Medical Sciences, Saifai, IND

**Keywords:** scapula, glenoid cavity, arthroplasty, glenoid notch, glenoid cavity index

## Abstract

Background and objective

The head of the humerus articulates with the glenoid cavity (GC) to form the shoulder joint. Understanding the various shapes and sizes of GC is important not only to analyse the stability of the glenohumeral joint but also to design prostheses for shoulder arthroplasty. Morphometric data on GC among the North Indian population is scarce. Hence, the aim of this study was to provide morphological and morphometric data on GC among the North Indian population.

Methods

This study was conducted in the department of anatomy of two medical colleges using undamaged dry scapulae. The shapes of GC and supero-inferior (SI) and maximum anteroposterior diameters above and below the notch were recorded. Statistical analysis and Student's t-test were carried out to identify statistically significant differences in diameters of the two sides of GC.

Results

The most common and least common shapes of GC were pear and inverted comma shapes respectively. The mean SI glenoid diameter was 33.6 ± 3.2 mm. The mean of H1 and H2 diameter was 23.6 ± 3.1 and 15.3 ± 2.1 mm respectively. The mean GC indices on the right and left sides were 72.16 and 68.14 respectively. In all of the above measurements, bilateral differences were not statistically significant (p-values of ˃0.05).

Conclusions

The morphometric data on GC may be used to design prostheses for shoulder arthroplasty among the North Indian population. The information is also useful in detecting various pathological conditions of the shoulder like rotator cuff disease, osteochondral defects, and Bankart lesions.

## Introduction

The scapula is a flat triangular bone situated posterolaterally on the thoracic cage spanning second to seventh ribs. Its lateral angle is truncated and is characterised by the presence of the glenoid cavity (GC), which articulates with the head of the humerus forming the glenohumeral joint. When the arm is by the side of the body, GC is directed forward, laterally, and slightly upwards. However, when the arm is above the head, it is directed straight upwards. Supero-inferiorly, it extends between the supra and infra glenoid tubercles [1]. A notch is present on its anterosuperior aspect, which is responsible for the different shapes of GC. When the glenoid notch is indistinct, GC appears piriform or pear-shaped; when it is distinct, it gives an inverted comma shape to GC. Moreover, when the glenoid notch is absent, GC appears oval in shape [2,3].

The long axis or vertical diameter of GC is broader below as compared to above and is longest. The glenohumeral joint is more prone to dislocation than other joints of the human body. Fractures and dislocations of GC are common.

It is reported in the literature that when the glenoid notch is distinct on the anterior margin of GC, the glenoid labrum is often not attached to the rim of GC at the site of the notch [3]. This may be a predisposing factor for anterior dislocation of the shoulder joint. The shape, size, height, and width are important anatomical parameters used to design prosthesis of GC [4]. Therefore, thorough knowledge of the variations of GC is of utmost importance in total shoulder arthroplasty.

To the best of our knowledge, there is no morphometric data on GC describing its morphology in the North Indian population, which would help develop and design prostheses of GC for total shoulder arthroplasty. This information will also be useful for treating shoulder pathological conditions like glenohumeral instability, rotator cuff pathology, osteochondral defects, and Bankart lesions [4]. The shape and size of GC vary in various races and also within the same population [5]. Hence, we conducted this study with the aim to provide data pertaining to the shapes and sizes of GC in the North Indian population.

## Materials and methods

A total of 172 dry assorted scapulae were observed in the anatomy departments of two medical colleges of North India for the present study. Out of 172 scapulae, 91 were right-sided and 81 left-sided. The age and sex of scapulae were not known. Supero-inferior (SI) and anteroposterior diameters of GC were measured by digital vernier calipers. The SI diameter was defined as the maximum distance between the supraglenoid tubercle and the most inferior point on the glenoid margin. For designing a more accurate prosthesis of GC, two dimensions, H1 and H2, of the maximum width below and above the notch of GC were observed.

Two indices of GC, namely GCI1 and GCI2, corresponding to diameters H1 and H2 have been defined for representing better aspect ratios of GC; so, GCI1 and GCI2 were formulated as follows:

GCI1 = anteroposterior diameter H1 x 100/SI diameter

GCI2 = anteroposterior diameter H2 x 100/SI diameter

Variables were expressed in terms of mean, standard deviation, and percentages. A paired t-test was used to compare the parameters of right-and left-sided GCs. P-values of <0.05 were considered statistically significant.

Scapulae that were fully ossified, dried, and complete in all respect were taken into account in this study as opposed to broken and unossified scapulae. Damaged/deformed or scapulae associated with pathologies like healed fractures were excluded from the study.

## Results

**Figure 1:**
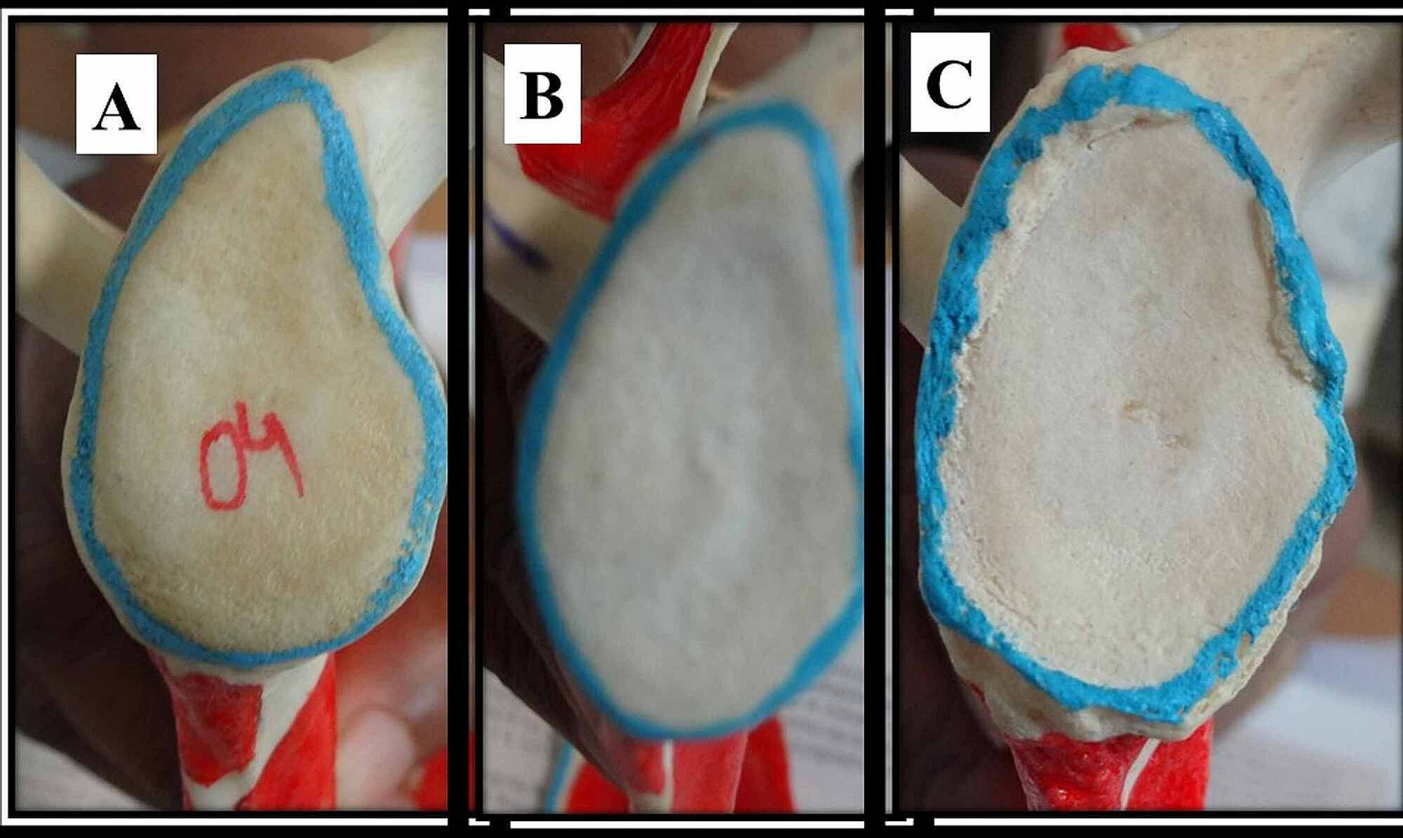
Various types of glenoid cavity A: comma-shaped. B: pear-shaped. C: oval-shaped

The most common shape of GC observed in the present study was pear shape (50%), followed by the oval shape (29.65%). The least common shape was the inverted comma shape (20.35%). The different shapes of GC are illustrated in Figure 1.

Detailed statistics related to different shapes of GC are presented in Table 1.

**Table 1:** Statistical analysis of various shapes of the glenoid cavity

Shape	Right, n (%)	Left, n (%)	Total, n (%)
Pear	45 (49.45%)	41 (50.62%)	86 (50%)
Oval	26 (28.57%)	25 (30.86%)	51 (29.65%)
Inverted comma	20 (21.98%)	15 (18.52%)	35 (20.35%)
Total	91 (100%)	81 (100%)	172 (100%)

The overall mean of SI glenoid diameters was 33.6 ± 3.2 mm. Right- and left-sided means of the SI, H1, and H2 are recorded in Table 2.

**Table 2:** Statistical analysis of various diameters of the glenoid cavity SI: supero-inferior diameter; HI: the maximum transverse diameter below the notch; H2: the maximum transverse diameter above the notch; SD: standard deviation

Diameter	Right range, mm	Left range, mm	Right mean ± SD, mm	Left mean ± SD, mm
SI	30-40	30-40	33.4 ± 3.0	33.9 ± 3.6
H1	20-36	20-30	24.1 ± 3.2	23.1 ± 2.9
H2	12-20	10-20	15.4 ± 2.0	15.3 ± 2.2

The overall means of H1 and H2 diameters were 23.6 ± 3.1 mm and 15.3 ± 2.1 mm respectively. The means of GCI1 on right and left sides were 72.16 and 68.14, and GCI2 means on right and left sides were 46.11 and 45.13 respectively. In all the above measurements, bilateral differences were not statistically significant (p-values of ˃0.05).

## Discussion

We carried out a morphometric analysis of GC and its significance in different groups of populations by direct measurement on embalmed cadavers, directly measuring dry scapulae, indirect radiographic measurements of scapulae harvested from the cadavers, and radiographic measurements in the living patients. The present study was conducted to measure the dimensions of GC on dry scapulae among the North Indian population.

GC is articulated with the humerus to form the glenohumeral joint. The morphometric parameters related to the shape and size of GC are very useful in the design and development of GC. These parameters are analysed below.

The shape of the glenoid cavity

In our study, the most common shape of GC was the pear shape, which is consistent with the observations of Mamatha et al. [6], Rajput et al. [7], Hassanein [8], and Sarwar et al. [9]; however, some researchers including El-Din and Ali [10] found the oval shape as the least common shape, which contrasts with our study where oval-shaped GC was observed as the second most common shape (Table 1). The inverted comma-shaped GC was observed as the second most common shape by the above scientists except El-Din and Ali [10], whereas the same was observed as the least common shape in our study; however, El-Din and Ali [10] found inverted comma-shaped GC as the most common and pear-shaped GC as the second most common type in the Egyptian population.

Prescher and Klümpen [3] reported only two types of GC, namely, pear-shaped in 55% and oval-shaped in 45% of the German population, which contrasts with studies of the present authors and other aforementioned scientists. Similarly, two types of GC (pear- and oval-shaped) were detected in a study on the Turkish population [11]. But the incidence of the most common oval-shaped variety of GC was 72% and that of the pear-shaped was 28% in the Turkish population [11].

Supero-inferior (SI) glenoid diameter

In our study, 33.6 ± 3.2 mm was the overall mean of SI glenoid diameter, which was lower than that in males and higher than that in females compared to the findings of Churchill et al. [2], Frutos [12], and Ozer et al. [13]; but it is closer to that in males and higher than that in females compared to the study by Patel et al. (Table 3) [14].

**Table 3:** Comparision of supero-inferior diameters of the glenoid cavity in studies by various authors SI: supero-inferior diameter; SD: standard deviation

Authors	SI mean ± SD, mm			
	Male	Female	Right side	Left side
Churchill et al. [2]	37.5 ± 2.2	32.6 ± 1.8	-	-
Frutos [12]	36.08 ± 2.0	31.7 ± 1.7	-	-
Ozer et al. [13]	38.71 ± 2.71	33.79 ± 3.08	-	-
Patel et al. [14]	34.64 ± 3.59	31.29 ± 1.64	-	-
Mamatha et al. [6]	-	-	33.67 ± 2.82	33.92 ± 2.87
Rajput et al. [7]	-	-	34.76 ± 3.0	34.43 ± 3.21
Kavita et al. [15]	-	-	35.2 ± 3.0	34.7 ± 2.8
Sarwar et al. [9]	-	-	35.22 ± 3.26	34.53 ± 3.21
El-Din and Ali [10]	-	-	38.88 ± 2.63	39.01 ± 2.49
Akhtar et al. [4]	-	-	36.03 ± 3.15	35.52 ± 3.12
Present study	-	-	33.4 ± 3.0	33.9 ± 3.6

The mean SI glenoid diameter of left shoulders was slightly higher than that of right shoulders in our study, which is similar to the findings of Mamatha et al. [6] and El-Din and Ali [10], but a contrasting trend was found by Akhtar et al. [4], Rajput et al. [7], Sarwar et al. [9] and Kavita et al. [15]. The mean SI glenoid diameter of right shoulders in our study is comparable with that of Mamatha et al. [6] but lower than that in Rajput et al. [7], Kavita et al. [15], El-Din and Ali [10], Sarwar et al. [9], and Akhtar et al. (Table 3) [4]. A similar trend was observed on the left side too (Table 3).

H1 diameter

The overall mean of H1 diameter in our study was comparable to that of females in the study by Churchill et al. [2], but it is higher in males of the above study, similar to the studies of Frutos [12] and Ozer et al. [13]. The sidewise comparison revealed that the H1 diameter of the right side in the present study was higher than the H1 diameter in the studies by Mamatha et al. [6], Rajput et al. [7], El-Din and Ali [10], Sarwar et al. [9], and Akhtar et al. [4] while it was lower than that reported by Kavita et al. (Table 4) [15].

**Table 4:** Comparison of H1 and H2 diameters of the glenoid cavity in studies by various authors H1: the maximum transverse diameter below the notch; H2: the maximum transverse diameter above the notch

Authors	H1, mm				H2, mm	
	Male	Female	Right side	Left side	Right side	Left side
Churchill et al. [2]	27.86 ± 1.6	23.6 ± 1.5	-	-	-	-
Frutos [12]	26.3 ± 1.5	22.31 ± 1.4	-	-	-	-
Ozer et al. [13]	27.33 ± 2.4	22.72 ± 1.72	-	-	-	-
Patel et al. [14]	23.89 ± 2.29	22.22 ± 2.73	-	-	-	-
Akhtar et al. [4]			23.67 ± 2.53	23.59 ± 2.47	16.30 ± 2.16	16 ± 2.34
Mamatha et al. [6]			23..35 ± 2.04	23.05 ± 2.30	16.27 ± 2.01	15.77 ± 1.96
Rajput et al. [7]			23.3 ± 3.0	22.92 ± 2.80	15.10 ± 2.54	13.83 ± 2.45
Sarwar et al. [9]			23.95 ± 2.78	23.64 ± 2.37	16.16 ± 2.38	15.34 ± 2.17
El-Din and Ali [10]			21.33 ± 2.49	21.69 ± 2.06	28.31 ± 2.38	27.99 ± 2.55
Kavita et al. [15]			25.07 ± 2.7	24.9 ± 2.0	16.8 ± 1.8	16.3 ± 2.0
Present study			24.1 ± 3.2	23.1 ± 2.9	15.4 ± 2.0	15.3 ± 2.2

On the left side, this diameter in our study was comparable to that observed by Mamatha et al. [6], Sarwar et al. [9], and Akhtar et al. [4]. The same was higher in our study than that detected by Rajput et al. [7] and El-Din and Ali [10].

H2 diameter

The mean H2 diameter (23 ± 2.7 mm) found in the study by Iannotti et al. [16] was higher compared to our study (15.3 ± 02.1 mm). On the right side, the mean H2 diameter in the studies of Mamatha et al. [6], Kavita et al. [15], El-Din and Ali [10], Sarwar et al. [9], and Akhtar et al. [4] is higher than that in the present study, and the same is lower in the study of Rajput et al. [7] as compared to our study.

On the left side, the H2 diameter in our study is comparable to that reported by Mamatha et al. [6] and Sarwar et al. [9], but higher than that in the study by Rajput et al. [7] and lower than that in the studies of Kavita et al. [15], El-Din and Ali [10], and Akhtar et al. (Table 4) [4].

Glenoid cavity index

GCI1 on the right and left side in the present study are 72.16% and 68.14% respectively. GCI (this GCI is the same as GCI1 in our case) on the right side as recorded by Akhtar et al. [4] (66.73%) and Dhindsa and Singh [17] (70.37%) is lower, while the same is higher in the study by Hassanein [8] (73.67%) compared to our study. On the left side, it is lower in the study by Akhtar et al. [4] (66.13%), but higher in the study by Hassanein [8] (76.71%) compared to our study. GCI2 on the right and left sides in our study are 46.11 and 45.13 respectively, and these have not been calculated by other scientists; hence, a comparison is not possible. GCI1 and GCI2 data will help in designing the glenoid component more accurately.

Clinical significance

Understanding the various shapes of GC and their morphometric parameters are not only useful in designing GC prosthesis for total shoulder arthroplasty but also important for analysing the stability of the glenohumeral joint. These anatomical variations of GC are clinically significant in identifying shape, size, and articular configuration for diagnostic interpretation of images of glenohumeral joint of the shoulder and access in the surgical procedure to restore mobility complications. The shape and morphology of the periphery of GC are crucial to analyse the attachment of labrum and depth of GC, which decide the stability of this joint. The glenoid labrum is either bridging the notch [3] present in inverted comma-shaped GCs, or the margin of the notch of GC remains unfastened, giving an appearance of labral tear, sublabral foramen, or Buford complex during arthroscopy. The sublabral foramen appears to form due to the congenital absence of the anterosuperior labrum and occurs in 12% of people [18].

A small recess of the joint cavity projecting between the glenoid labrum and the anterior margin of GC makes the shoulder joint more likely to dislocate, resulting in labral tear and avulsions at the anterior margin of the GC [3]. The oval-shaped GC is the most stable type as the glenoid labrum is attached all along the borders of GC [5]. As the relative risk of shoulder joint instability is maximum in inverted comma-shaped GC, less likely in the pear-shaped GC, and least likely and most stable in the oval-shaped one. In our case, the most common shape was the pear shape followed by the oval shape, and the inverted comma was the least common shape. It indicates that this segment of the population is least vulnerable to anterior shoulder dislocation. However, the glenohumeral joint is subject to more anti-stability forces among sports personnel as this shoulder joint is used more vigorously during shotput and javelin throws, badminton, table and lawn tennis hits, volleyball shots, batting in cricket, etc.; so they are more prone to shoulder joint dislocation/injury.

The morphometry of GC helps in the analysis of frequent dislocation of the glenohumeral joint and complications of rotator cuff disease [11,19]. The procedures for total shoulder arthroplasty also suffer from complexities when they encounter anatomical variants like bony defects of the anterior and/or posterior margins of GC. These defects lead to incomplete restorations of this joint [2,11].

## Conclusions

We believe that the data on GC provided in this study will be of utmost use in constructing prostheses in cases where right and left shoulders separately requiring total shoulder arthroplasty in the North Indian population. The data will also be of great value in assessing various pathological conditions like rotator cuff disease, osteochondral defects, and Bankart lesions. Knowledge of the shape and morphometry of the glenoid fossa is essential for treating glenohumeral osteoarthritis. The shape and size of GC not only varies with ethnicity among different races but also within the same population.

Also, the morphometric data on GC in the present study will be useful to orthopaedic practitioners and prosthetists for designing glenoid prostheses for the North Indian population, and also to anthropologists in their analysis of the evolution of the bipedal gait.
